# Association of nasopharynx cancer with obstructive sleep apnea

**DOI:** 10.3389/fonc.2026.1880900

**Published:** 2026-07-20

**Authors:** Yi-Juan Huang, Shih-Han Hung, Hsin-Chien Lee, Herng-Ching Lin, Shiu-Dong Chung, Chin-Shyan Chen

**Affiliations:** 1 School of Health Care Administration, College of Management, Taipei Medical University, Taipei, Taiwan; 2 Department of Otolaryngology, Taipei Medical University, Taipei, Taiwan; 3 Department of Otolaryngology, Mennonite Christian Hospital, Hualien, Taiwan; 4 International Ph.D. Program in Medicine, College of Medicine, Taipei Medical University, Taipei, Taiwan; 5 Graduate Institute of Clinical Psychology, College of Medicine, Taipei Medical University, Taipei, Taiwan; 6 Department of Psychiatry & Sleep Center, Taipei Medical University Hospital, Taipei, Taiwan; 7 Research Center of Data Science on Healthcare Industry, College of Management, Taipei Medical University, Taipei, Taiwan; 8 Division of Urology, Department of Surgery, Far Eastern Memorial Hospital, Banciao, New Taipei, Taiwan; 9 Department of Nursing, College of Healthcare & Management, and General Education Center, Asia Eastern University of Science and Technology, New Taipei, Taiwan; 10 Department of Economics, National Taipei University, New Taipei, Taiwan

**Keywords:** epidemiology, head & neck cancers, head & neck carcinoma, nasopharyngeal carcinoma, obstructive sleep apnea

## Abstract

**Introduction:**

Despite the extensive research conducted on the association between OSA and HNCs, the specific relationship between obstructive sleep apnea (OSA) and nasopharyngeal carcinoma (NPC) has received comparatively less attention. The present study aimed to explore the association between OSA and NPC.

**Methods:**

The data for this study was drawn from the Longitudinal Health Insurance Database 2010. The study incorporated a total of 3,235 NPC patients and 12,940 propensity score-matched controls. The NPC and controls’ baseline clinical and demographic parameters were compared using standardized mean difference. A conditional logistic regression model was developed in order to quantify the association between OSA and the odds of NPC.

**Results:**

The prevalence of prior OSA was significantly higher in the NPC group than in the control group (2.07% vs. 1.51%, p = 0.023). Following the adjustment for a comprehensive set of confounders, including age, sex, income, geography, urbanization, diabetes, hypertension, hyperlipidemia, COPD, chronic rhinitis, sinusitis, human papillomavirus infection, tobacco use disorder, and alcohol-related disorders, pre-existing OSA remained associated with an increased odds of developing NPC (adjusted OR = 1.42, 95% CI = 1.07, 1.89). In sex-stratified analysis, the point estimates for the association between OSA and NPC were similar for both males (adjusted OR = 1.41, 95% CI = 1.03, 1.94) and females (adjusted OR = 1.46, 95% CI = 0.77, 2.78). While the association only reached statistical significance in the males, the lack of significance in females may be indicative of lower statistical precision due to the smaller sample size.

**Conclusion:**

The present study identifies an association between prior OSA diagnosis and NPC, particularly in the males. Further investigation is required to confirm this relationship and its underlying mechanisms.

## 1 Introduction

Nasopharyngeal carcinoma (NPC) is a distinctive form of head and neck cancer that exhibits a distinct geographical distribution, predominantly prevalent in Southeast Asia and Southern China. The etiology of the condition is multifactorial, involving a combination of genetic susceptibility, environmental influences, and a notable association with Epstein-Barr virus (EBV) infection (1). Notwithstanding the advances in treatment options, NPC continues to present a considerable health challenge, frequently diagnosed at advanced stages due to its occult location and nonspecific early symptoms. The long-term prognosis may be influenced by treatment-related adverse effects and the potential for subsequent recurrence.

Obstructive sleep apnea (OSA) is a prevalent sleep condition characterized by recurrent upper airway obstruction during sleep, resulting in transient oxygen deficiency and disrupted sleep (2, 3). The global prevalence of OSA is increasing, driven by rising obesity rates and an aging population (4, 5). In addition to the established cardiovascular and metabolic consequences (2, 6, 7), recent evidence suggests a potential association between OSA and the development and progression of cancer (8–14). It has been hypothesized that the intermittent hypoxia and persistent inflammation associated with OSA may contribute to the establishment of a pro-tumorigenic microenvironment, exerting a multifaceted influence on various aspects of cancer biology, including angiogenesis, metastasis, and immune evasion (15–17).

The association between OSA and various forms of cancer has garnered increasing attention in recent years. A number of systematic reviews and meta-analyses have investigated the broader relationship between OSA and general cancer risk, with some research suggesting a heightened cancer incidence in patients with OSA (8, 9, 17). In the context of head and neck cancers (HNCs), OSA is recognized as a significant comorbidity, often exacerbated by anatomical alterations induced by the tumor or by treatment modalities such as radiotherapy (18, 19). The anatomical proximity of the nasopharynx to the upper airway, which is directly influenced by OSA, suggests a probable direct connection. Nasopharyngeal tumors have been observed to result in or exacerbate OSA by obstructing the airway. Conversely, the persistent inflammation and tissue changes caused by OSA have the potential to render the nasopharyngeal epithelium more susceptible to cancerous changes, particularly in those with additional risk factors such as EBV infection (1).

The pathophysiology of OSA is characterized by a series of interconnected mechanisms, including intermittent hypoxia, activation of the sympathetic nervous system, and systemic inflammation (20). Despite the extensive research conducted on the association between OSA and HNCs, the specific relationship between OSA and NPC has received comparatively less attention (18, 19). In consideration of the distinctive characteristics of NPC, including its distinct etiology and anatomical location, 1 a dedicated investigation into its association with OSA is indicated. The present study aims to evaluate the association between OSA and the odds of NPC using a large, population-based case-control design, thereby providing further insights into this complex relationship and its potential clinical implications.

## 2 Methods

### 2.1 Data source and ethical considerations

This retrospective, case-control study utilized data from the Longitudinal Health Insurance Database 2010 (LHID2010), a nationally representative cohort database derived from Taiwan’s National Health Insurance (NHI) program. The NHI program, a single-payer system that was initiated in 1995, provides universal healthcare coverage. The LHID2010 is a valuable instrument for epidemiological research, as it contains all of the anonymized registration and medical claims information for two million beneficiaries who were selected at random from the NHI registry. The present study was ethically reviewed by the Research Ethics Committee of National Taiwan University (approval number: 202312EM054), in accordance with the principles of the Declaration of Helsinki. The requirement for patient informed consent was waived by the Research Ethics Committee of National Taiwan University on the grounds that all personal identifiers had been deleted from the database and the study was retrospective in nature.

### 2.2 Study population and group matching

The case group consisted of 3,235 individuals aged 18 years or older who received a new diagnosis of NPC, identified by ICD-9-CM code 147 or ICD-10-CM code C11, between 1 January 2010 and 31 December 2020. The initial NPC diagnosis date was designated as the index date for each case.

A control group was selected from the remaining LHID2010 beneficiaries who were at least 18 years old and had no prior history of NPC. In order to minimize selection bias, a 1:4 propensity score matching (PSM) technique was employed. The propensity score for each individual in the case and potential control pools was calculated via a non-parsimonious logistic regression model. In this model, the binary dependent variable was the NPC group status (case vs. control), and the independent variables encompassed a comprehensive range of baseline covariates: age, gender, socioeconomic status (monthly income, geographic location, and residential urbanization level), and all pre-existing comorbidities (diabetes, hypertension, hyperlipidemia, COPD, chronic rhinitis, sinusitis, HPV infection, tobacco use disorder, and alcohol-related disorders). The monthly income of the subjects was categorized into three groups based on the insurance premium levels at the index year: (<NT$15,841; NT$15,841–25,000; ≥NT$25,001). Urbanization levels were defined according to the National Health Research Institutes’ classification, which takes into account population density and socioeconomic development. The presence of all comorbidities, including but not limited to diabetes, hyperlipidemia, and COPD, was ascertained by the presence of the respective ICD codes in at least one inpatient claim or two outpatient claims within the year prior to the index date. This ensured that the comorbidities were categorized as pre-existing conditions. Each NPC patient was then matched to four controls using a nearest-neighbor random matching algorithm with a caliper width of 0.2. The index date for controls was assigned to correspond with the calendar year of their corresponding case’s diagnosis. The final study cohort comprised 3,235 NPC patients and 12,940 matched controls.

### 2.3 Ascertainment of obstructive sleep apnea

The primary exposure of interest, OSA, was ascertained from ambulatory care claims filed between 2005 and 2020. An individual was classified as having OSA if their records contained a relevant diagnostic code (ICD-9-CM: 327.23, 780.51, 780.53, 780.57; ICD-10-CM: G47.30, G47.33). In order to enhance the diagnostic accuracy, an OSA diagnosis was considered valid only if substantiated by a record of a polysomnography procedure (procedure code: 89.17; DRUG_NO codes: 17008A/17008B). Moreover, the present study exclusively encompasses OSA cases that were diagnosed prior to the designated index date. In this study, OSA was treated as a binary exposure variable (yes/no), defined on the basis of the presence of relevant ICD diagnostic codes and a corresponding polysomnography procedure record prior to the index date. Due to the absence of physiological data such as the Apnea-Hypopnea Index (AHI) in the administrative database, we were unable to assess the severity of OSA or evaluate a potential dose-response relationship with NPC.

### 2.4 Statistical analysis

All statistical analyses were carried out using SAS software (version 9.4, SAS Institute Inc., Cary, NC). The NPC and controls’ baseline clinical and demographic parameters were assessed using standardized mean difference (SMD), with a threshold of <0.1 indicating satisfactory balance. To account for the 1:4 matched case-control design, conditional logistic regression was performed using the matched sets as strata. This analytical approach successfully controls for the statistical dependency between cases and their respective matched controls, ensuring that the estimated standard errors and confidence intervals correctly reflect the matched architecture of the data. Furthermore, a ‘doubly robust’ estimation approach was executed by re-entering all baseline covariates simultaneously back into the conditional logistic regression model to further adjust for any residual confounding or minor post-matching imbalances. This strategy aims to correct for any residual imbalances (e.g., urbanization level) and to provide more precise and less biased estimates of the association between OSA and NPC. Furthermore, a prespecified sex-stratified analysis was conducted to determine if the association between prior OSA and NPC varied by sex, considering the distinct prevalence rates of both conditions in male and female populations. The association was measured using odds ratios (ORs) and 95% confidence intervals (CIs). A two-sided p-value less than 0.05 was considered to be statistically significant in all analyses.

## 3 Results

The baseline socio-demographic characteristics and comorbidities of the NPC cases and their matched controls are presented in **Table 1**. Following the implementation of propensity score matching, the two cohorts demonstrated a satisfactory balance, exhibiting no statistically significant disparities in mean age (SMD ≤ 0.001), sex (SMD ≤0.001), monthly income (SMD = 0.043), or geographic distribution (SMD = 0.020). However, a significant difference remained in urbanization levels (SMD = 0.118), which was further adjusted for in the multivariable analysis. Furthermore, the prevalence of all evaluated comorbidities, including diabetes, hypertension, hyperlipidemia, COPD, chronic rhinitis, sinusitis, HPV infection, tobacco use disorder, and alcohol-related disorders, was comparable between the groups (all SMD<0.001).

**Table 1:** Demographic characteristics of patients diagnosed with nasopharyngeal carcinoma and propensity score-matched controls (n=16,175).

Variable	Patients with nasopharyngeal carcinoma		Propensity score-matched controls		Standardized mean difference
	(n=3235)		(n=12,940)		
	Total no.	%	Total no.	%	
Age, mean (SD)	51.7	14.7	51.7	14.7	<0.001
Males	2060	63.7	8240	63.7	<0.001
Monthly Income					0.043
<NT$1~15,841	582	18.0	2114	16.3	
NT$15,841~25,000	1156	35.7	4630	35.8	
≥NT$25,001	1497	46.3	6196	47.9	
Geographic region					0.020
Northern	1470	45.4	6012	46.5	
Central	811	25.1	3190	24.7	
Southern	863	26.7	3415	26.4	
Eastern	91	2.8	323	2.5	
Urbanization level					0.118
1 (most urbanized)	921	28.5	3781	29.2	
2	950	29.4	4084	31.6	
3	538	16.6	2181	16.9	
4	486	15.0	1883	14.6	
5 (least urbanized)	340	10.5	1011	7.8	
Hypertension	1382	42.7	5528	42.7	<0.001
Hyperlipidemia	1269	39.2	5076	39.2	<0.001
Diabetes	791	24.5	3164	24.5	<0.001
HPV infection	252	7.8	1008	7.8	<0.001
COPD	431	13.3	1724	13.3	<0.001
Chronic rhinitis	1083	33.5	4332	33.5	<0.001
Sinusitis	2435	75.3	9740	75.3	<0.001
Tobacco use disorder	156	4.8	624	4.8	<0.001
Alcohol abuse	15	0.5	60	0.5	<0.001

A total of 67 patients in the NPC group (2.07% of 3,235) and 195 individuals in the control group (1.51% of 12,940) had a prior diagnosis of OSA (p=0.023), as detailed in **Table 2**. Following the adjustment for a comprehensive set of potential confounders in a conditional logistic regression model, pre-existing OSA remained independently associated with an increased odds of developing NPC (adjusted OR = 1.42, 95% CI = 1.07, 1.89). Individuals with a history of OSA had 1.42 times the odds of an NPC diagnosis.

**Table 2:** Prevalence rates and odds ratio of obstructive sleep apnea among patients diagnosed with nasopharyngeal carcinoma in comparison with controls.

Presence of OSA	Total (N = 16,175)		Patients with nasopharyngeal carcinoma (n=3235)		Controls (n=12,940)	
	n, %					
Yes	262	1.6	67	2.1	195	1.5
No	15,913	98.4	3168	97.9	12,745	98.5
Odds ratio ^a^ (95% CI)	1.42 (1.07, 1.89)					

^a^Adjusted for age, sex, monthly income, geographic location, urbanization level, hyperlipidemia, diabetes, hypertension, chronic rhinitis, sinusitis, COPD, HPV infection, tobacco use disorder, and alcohol-related disorders; Odds ratios were estimated using conditional logistic regression to account for the 1:4 matched study design.

In sex-stratified analysis (see **Table 3**), the point estimates for the association between OSA and NPC were similar for both males (adjusted OR = 1.41, 95% CI = 1.03, 1.94) and females (adjusted OR = 1.46, 95% CI = 0.77, 2.78). While the association only reached statistical significance in the male participants, a comparable yet non-significant association was identified among the female participants.

**Table 3:** Prevalence rates and odds ratio of obstructive sleep apnea among patients diagnosed with nasopharyngeal carcinoma in comparison with controls according to sex.

Males						
Presence of OSA	Total (N = 10,300)		Patients with nasopharyngeal carcinoma (n=2060)		Controls (n=8240)	
	n, %					
Yes	213	2.1	54	2.6	159	1.9
No	10,087	97.9	2006	97.4	8081	98.1
Odds ratio ^a^ (95% CI)	1.41 (1.03, 1.94)					
Females						
Presence of OSA	Total (N = 5875)		Patients with nasopharyngeal carcinoma (n=1175)		Controls (n=4700)	
	n, %					
Yes	49	0.8	13	1.1	36	0.8
No	5826	99.2	1162	98.9	4664	99.2
Odds ratio ^a^ (95% CI)	1.46 (0.77, 2.78)					

^a^Adjusted for age, monthly income, geographic location, urbanization level, hyperlipidemia, diabetes, hypertension, chronic rhinitis, sinusitis, COPD, HPV infection, tobacco use disorder, and alcohol-related disorders; Odds ratios were estimated using conditional logistic regression to account for the 1:4 matched study design.

## 4 Discussion

This large-scale, population-based case-control study provides modest evidence for an independent association between OSA and an increased odds of NPC. Our findings, derived from a robust propensity score-matched cohort within Taiwan’s National Health Insurance Database, demonstrate that individuals with a prior diagnosis of OSA had a significantly higher likelihood of developing NPC, with an adjusted odds ratio of 1.42 (95% CI = 1.07, 1.89). This association was particularly pronounced in males, where a significant increase in NPC odds was observed among those with a history of OSA.

The present findings are consistent with a growing body of literature suggesting a broader link between OSA and various cancers (8, 9, 17). The intermittent hypoxia (IH) and chronic inflammation that are characteristic of OSA are widely hypothesized to create a pro-tumourigenic microenvironment (15, 16, 21). It has been established that increased oxidative stress, angiogenesis and epithelial-mesenchymal transition are all critical processes in the initiation and progression of cancer (15, 21–23). The release of pro-inflammatory cytokines can also promote cell proliferation, inhibit apoptosis, and facilitate metastatic spread. The systemic effects of OSA have been demonstrated to have the potential to contribute to the development of malignancies in various organs, including those in the head and neck region.

A substantial body of research has previously indicated an elevated cancer risk in OSA patients (8, 9, 12, 13, 17). For instance, a meta-analysis demonstrated that individuals with OSA exhibited a risk ratio for developing cancer that was 40% to 50% higher (8). While these studies frequently encompass a broad spectrum of cancer types, the present study is specifically focused on NPC, a malignancy that exhibits unique epidemiological and etiological characteristics. The anatomical proximity of the nasopharynx to the upper airway, which is directly affected by OSA, suggests a plausible direct link.

Nasopharyngeal tumors have been observed to cause or exacerbate OSA by obstructing the airway. Conversely, the chronic inflammatory state and tissue remodeling induced by OSA might predispose the nasopharyngeal epithelium to malignant transformation, especially in individuals with other risk factors like EBV infection (1).

The observed point estimates appear similar between sexes (adjusted OR = 1.41 for males and 1.46 for females); however, due to the much wider confidence interval in the female cohort (95% CI = 0.77, 2.78), these findings must be interpreted with caution as they present low statistical precision. While the lack of statistical significance in females might be related to the smaller sample size, the wide confidence interval means we cannot definitively rule out a true biological difference or a lack of real association within the female population. For instance, males generally exhibit a higher prevalence and more severe forms of OSA compared to females (10–12), which could theoretically lead to a greater cumulative exposure to the pro-tumorigenic effects of IH and inflammation (2, 4). Therefore, the exact nature of this sex disparity remains inconclusive and requires clearer validation in future prospective cohorts.

### 4.1 Molecular mechanisms underlying the OSA-NPC association

The observed association between OSA and NPC can be further elucidated by considering the intricate molecular mechanisms through which intermittent hypoxia (IH) and systemic inflammation, hallmarks of OSA, contribute to carcinogenesis. It is widely accepted that IH is a defining feature of OSA. This condition is characterized by repetitive cycles of oxygen desaturation and reoxygenation. This fluctuating oxygen environment has been demonstrated to be a potent inducer of cellular stress, leading to the generation of reactive oxygen species (ROS) (21–24). Elevated ROS levels have been demonstrated to induce DNA damage, genomic instability, and mutations, thereby instigating or promoting cancer development (21–24). Furthermore, IH has been demonstrated to activate hypoxia-inducible factors (HIFs), with a particular emphasis on HIF-1α, which have been identified as the primary regulatory elements governing cellular responses to hypoxia. HIF-1α has been demonstrated to promote a number of crucial processes for tumor growth and metastasis, including angiogenesis, glycolysis and cell proliferation (15, 17). In the context of NPC, the activation of HIF-1α by IH has been demonstrated to accelerate tumor progression and enhance its invasive potential (25).

In addition to its direct impact on cellular structures, IH exerts a significant influence on the tumor microenvironment. It has been demonstrated that this process can induce the expression of various pro-angiogenic factors, including vascular endothelial growth factor (VEGF), thus facilitating the formation of new blood vessels that supply nutrients and oxygen to rapidly growing tumors (15, 17, 26). This enhanced angiogenesis is critical for tumor expansion and metastatic dissemination. Furthermore, IH has been demonstrated to modulate immune responses, thereby engendering an immunosuppressive environment that fosters tumor escape from immune surveillance. For instance, intermittent hypoxia has been demonstrated to enhance the expression of programmed death-ligand 1 (PD-L1) on tumor cells, thereby impeding anti-tumor T-cell responses (17). Furthermore, alterations in tumor-associated macrophages (TAMs) by IH also contribute to a pro-tumorigenic milieu, promoting tumor growth and metastasis (26).

OSA-induced vascular dysfunction represents a further critical pathway through which OSA may lead to cancer development. The chronic intermittent hypoxia that is characteristic of OSA leads to endothelial dysfunction and vascular remodeling, which in turn affects microvascular integrity throughout the body, including in the nasopharyngeal region (27). These vascular changes have the potential to compromise the integrity of tissue barriers and promote abnormal angiogenesis, thereby creating a microenvironment conducive to malignant transformation. The retinal vasculature, which has been demonstrated to exhibit similar pathophysiological responses to hypoxia as other vascular beds, has been shown to exhibit measurable changes in OSA patients, reflecting broader systemic vascular dysfunction (27). Such vascular alterations in the nasopharyngeal region could facilitate tumor initiation and progression.

It is evident that another key consequence of OSA is chronic systemic inflammation, which has been demonstrated to play a pivotal role in cancer development. The recurrent episodes of IH and sleep fragmentation in OSA trigger a persistent low-grade inflammatory state, characterized by elevated levels of pro-inflammatory cytokines such as TNF-α, IL-6, and C-reactive protein (CRP) (7, 19, 28). These inflammatory mediators have been demonstrated to directly promote cell proliferation, inhibit apoptosis, and stimulate angiogenesis. Furthermore, they have been observed to activate various signaling pathways, including NF-κB and STAT3, which are frequently dysregulated in cancer and contribute to tumor cell survival and resistance to therapy. In non-small cell lung cancer (NSCLC), chronic inflammation, often exacerbated by EBV infection, is a known driver of tumourigenesis. The synergistic effect of OSA-induced inflammation and EBV-mediated inflammatory responses could create a particularly fertile ground for NPC development and progression.

In addition, OSA has been associated with metabolic dysregulation, encompassing insulin resistance and altered lipid metabolism, which are also recognized as risk factors for various cancers (2, 28). While the direct link to NPC requires further investigation, these systemic metabolic changes induced by OSA could indirectly contribute to a pro-cancer environment. The interplay between IH, inflammation, and metabolic alterations creates a complex network of pathways that collectively promote carcinogenesis in OSA patients (29).

### 4.2 Clinical implications and therapeutic considerations

The modest association between OSA and NPC identified in this study offers insightful directions for future clinical research. Given the high prevalence of OSA in the general population and the endemic nature of NPC in certain regions, the findings of this study suggest that OSA could represent a potential associated factor or a comorbidity worthy of exploration regarding its role in NPC prognosis. While the retrospective nature of our data limits direct clinical application, these findings primarily serve as hypothesis generation for prospective trials to evaluate whether managing OSA could influence patient outcomes. Instead of mandating immediate changes in clinical screening protocols, our results highlight a potential area for future cohort studies to observe the long-term clinical trajectory of patients with both conditions. Conversely, while patients diagnosed with OSA and severe IH might be tracked in future prospective settings, our current data underscore the need for targeted prospective trials to clarify whether specialized monitoring for head and neck malignancies is warranted, rather than justifying immediate increased clinical surveillance based on this single observation.

Therapeutic interventions for OSA, primarily Continuous Positive Airway Pressure (CPAP), have been shown to effectively mitigate IH and improve sleep quality (2, 3). The question then arises of whether CPAP therapy, by alleviating the pro-tumorigenic effects of OSA, could reduce the risk of NPC development or improve the prognosis in NPC patients with coexisting OSA. Despite the absence of specific evidence directly linking CPAP to reduced NPC incidence, studies on other cancers have demonstrated encouraging results. For instance, research suggests that CPAP treatment may improve cancer-specific survival in patients with OSA and cancer (13). This underscores a pivotal domain for prospective research, notably randomized controlled trials, to assess the impact of OSA treatment on NPC incidence and outcomes. Beyond the scope of CPAP, other OSA treatments, including oral appliances, positional therapy, and surgical interventions, also merit investigation within this paradigm (29).

Moreover, the presence of OSA in NPC patients has the potential to complicate treatment and recovery. Patients undergoing radiotherapy for NPC frequently experience anatomical changes in the upper airway, which can exacerbate pre-existing OSA or induce new-onset OSA (9, 14). Unmanaged OSA during and after cancer treatment has been demonstrated to result in increased fatigue, reduced quality of life, and potentially compromised adherence to cancer therapy. Consequently, a multidisciplinary approach involving sleep specialists, oncologists, and otolaryngologists is imperative for comprehensive patient care. The integration of OSA screening and management into the standard care pathway for NPC patients has the potential to optimize treatment outcomes and enhance overall well-being.

The present study utilizes a substantial, population-based dataset, thereby minimizing selection bias through the implementation of propensity score matching and the adjustment of a comprehensive set of confounders, encompassing demographic factors, comorbidities, and lifestyle/viral factors. The robust methodology employed in this study serves to enhance the generalizability and validity of the findings. The utilization of ICD codes in conjunction with Polysomnography Procedure codes for the identification of OSA enhances diagnostic accuracy, thereby addressing a prevalent limitation observed in studies that employ solely diagnostic codes (3).

Notwithstanding the study’s merits, it is important to acknowledge its limitations. Firstly, as a retrospective study utilizing administrative claims data, the accuracy and completeness of coding represents a limitation of the study. Despite the endeavor to enhance the accuracy of OSA diagnosis, it is acknowledged that some cases may be misclassified. Furthermore, a notable limitation of this study is the reliance on administrative codes (ICD-9-CM: 147; ICD-10-CM: C11) for NPC identification without access to the ICD-O-3 classification. This lack of histological and staging data means that our case group might include a heterogeneous mix of nasopharyngeal malignancies, including lymphomas or rare neuroendocrine tumors, rather than exclusively keratinizing or non-keratinizing squamous cell carcinomas. However, given that squamous cell carcinoma represents the overwhelming majority of nasopharyngeal malignancies in Taiwan, the impact of this misclassification on our primary findings is likely minimal. Nevertheless, the absence of tumor stage information prevents us from assessing whether OSA is associated with more advanced stages of NPC at diagnosis.

Secondly, this study faces the potential for residual confounding due to the absence of several key risk factors in the administrative database. Specifically, information on EBV infection status—the primary etiologic driver of NPC—was unavailable. Similarly, detailed lifestyle factors, such as dietary habits (e.g., consumption of salted fish or preserved foods), specific smoking intensity (pack-years), and alcohol consumption patterns, were not captured. Furthermore, although obesity is highly relevant to OSA, the LHID2010 does not record Body Mass Index. While we attempted to adjust for obesity-related conditions like diabetes and hypertension as proxies, these unmeasured variables may lead to residual confounding, potentially influencing the observed association between OSA and NPC.

Thirdly, the study’s design demonstrates an association rather than definitive causality, as capturing the precise chronological timeline on an individual level remains challenging in retrospective administrative databases. Both OSA and NPC possess insidious and progressive courses, meaning the recorded diagnosis date reflects clinical recognition rather than true biological onset. Consequently, our conclusions face potential threat from protopathic bias, where an undetected nasopharyngeal tumor might physically obstruct the airway and induce secondary sleep apnea prior to the formal cancer diagnosis. While a lag-time sensitivity analysis could theoretically evaluate this, further lagging the exposure window in our study was mathematically unfeasible; given that only 67 NPC patients presented with prior OSA, truncating the timeline would drastically reduce the sample size, severely strip the model of statistical power, and yield highly unstable estimates. To mitigate this inherent limitation, we strictly required all OSA exposures to be confirmed by polysomnography procedures before the index date. Nevertheless, due to these constraints, our findings must be interpreted strictly as hypothesis-generating for future prospective cohorts rather than definitive proof of a sequential causal pathway.

Fourthly, as in most claim-based studies, the identification of OSA was based on the diagnostic codes contained in the administrative data. It is well recognized that a significant proportion of OSA patients in the general population remain undetected. Consequently, the prevalence of OSA found in claim databases is usually lower than the prevalence reported in population-based epidemiological studies. This underdiagnosis may have resulted in the inclusion of some undiagnosed OSA patients in the control group. Such a non-differential misclassification is expected to bias the estimated association to zero and may therefore lead to underestimation of the true association between OSA and NPC. As was also the case in the findings of earlier studies based on claims which were carried out in other populations, including in Korea, similar patterns were observed. In order to address this limitation, a record of the polysomnography procedure has been requested in order to enhance the validity of the OSA diagnosis. Nevertheless, the possibility of residual misclassification cannot be entirely excluded and should be considered when interpreting the findings.

Finally, another potential source of bias is surveillance bias (or detection bias). Since OSA patients frequently experience upper airway symptoms, they are more likely to seek consultation from otolaryngologists, which may lead to a higher rate of incidental or earlier detection of NPC compared to individuals without OSA. To rigidly account for this variation in healthcare visits, we utilized chronic rhinitis and sinusitis—two primary drivers for ENT clinical visits—as clinical proxies within our propensity score matching framework. Following matching, both cohorts achieved a perfect balance in these conditions (33.5% for rhinitis and 75.3% for sinusitis, both SMD < 0.001), indicating an identical baseline exposure to ENT specialists and clinical examinations. While the possibility of residual surveillance bias cannot be entirely eliminated in any claims-based study, this rigorous balancing of ENT-specific healthcare utilization strongly minimizes its confounding effect on the observed independent association.

### 4.3 Future research directions

The present study opens several avenues for future research. Firstly, the establishment of prospective cohort studies is required in order to ascertain a definitive causal link and temporal relationship between OSA and NPC. Such studies could also investigate the dose-response relationship between OSA severity and NPC risk. Secondly, further mechanistic studies are essential to fully unravel the molecular pathways involved. This includes detailed investigations into the specific roles of various inflammatory mediators, immune cell populations, and genetic factors in mediating the OSA-NPC association. The observed sex disparity also necessitates research into hormonal influences, genetic differences, and sex-specific lifestyle factors that might explain the stronger association in males.

Thirdly, the importance of interventional studies, in particular randomized controlled trials, in determining the efficacy of OSA treatment, such as CPAP, in reducing the incidence of NPC or enhancing prognosis, cannot be overstated. Furthermore, these trials could explore the impact of OSA management on treatment-related toxicities and quality of life in NPC patients. Fourthly, the development of predictive biomarkers for NPC risk in OSA patients, or for OSA severity in NPC patients, would be highly valuable for personalized medicine approaches. Finally, further research into the interaction between OSA and other well-established NPC risk factors, such as EBV infection and environmental exposures, could provide a more comprehensive understanding of NPC etiology and inform targeted prevention strategies.

In summary, our study demonstrates a modest epidemiological link between pre-existing OSA and NPC. Rather than demonstrating a direct causal pathway, these findings should be viewed as hypothesis-generating, establishing a foundation for future prospective trials and mechanistic studies to clarify the true clinical impact of sleep apnea on nasopharyngeal oncogenesis.

## Data Availability

The data analyzed in this study is subject to the following licenses/restrictions: The datasets generated and analyzed during the current study are not publicly available due to the privacy protection regulation but are available from the corresponding author on reasonable request. Requests to access these datasets should be directed to Health and Welfare Data Centre, Ministry of Health and Welfare, Taiwan.

## References

[1] Chen YP Chan ATC Le QT Blanchard P Sun Y Ma J . Nasopharyngeal carcinoma. Lancet. (2019) 394:64–80. doi: 10.1016/0030-4220(93)90436-8 31178151

[2] Gottlieb DJ Punjabi NM . Diagnosis and management of obstructive sleep apnea: a review. JAMA. (2020) 323:1389–400. doi: 10.1001/jama.2020.3514 32286648

[3] Epstein LJ Kristo D Strollo PJ Jr Friedman N Malhotra A Patil SP . Clinical guideline for the evaluation, management and long-term care of obstructive sleep apnea in adults. J Clin Sleep Med. (2009) 5:263–76. doi: 10.5664/jcsm.27497 PMC2699173 19960649

[4] Young T Palta M Dempsey J Skatrud J Weber S Badr S . The occurrence of sleep-disordered breathing among middle-aged adults. N Engl J Med. (1993) 328:1230–5. doi: 10.1056/nejm199304293281704 8464434

[5] Peppard PE Young T Palta M Skatrud J . Longitudinal study of moderate weight change and sleep-disordered breathing. JAMA. (2000) 284:3015–21. doi: 10.1001/jama.284.23.3015 11122588

[6] Gozal D Kheirandish-Gozal L . Cardiovascular morbidity in obstructive sleep apnea: oxidative stress, inflammation, and much more. Am J Respir Crit Care Med. (2008) 177:369–75. doi: 10.1164/rccm.200608-1190pp 17975198 PMC2258438

[7] Lévy P Pépin JL Arnaud C Tamisier R Borel JC Dematteis M . Intermittent hypoxia and sleep-disordered breathing: current concepts and perspectives. Eur Respir J. (2008) 32:1082–95. doi: 10.1183/09031936.00013322 18827154

[8] Wu D Zhao Z Chen C Lu G Wang C Gao S . Impact of obstructive sleep apnea on cancer risk: a systematic review and meta-analysis. Sleep Breath. (2023) 27:843–52. doi: 10.1007/s11325-022-02695-y 36129602

[9] Kendzerska T Leung RS Hawker G Tomlinson G Gershon AS . Obstructive sleep apnea and the prevalence and incidence of cancer. CMAJ. (2014) 186:985–92. doi: 10.1503/cmaj.140238 25096668 PMC4162778

[10] Sillah A Watson NF Schwartz SM Gozal D Phipps AI . Sleep apnea and subsequent cancer incidence. Cancer Causes Control. (2018) 29:987–94. doi: 10.1007/s10552-018-1073-5 30120643 PMC6162141

[11] Brenner R Kivity S Peker Y Reinhorn D Keinan-Boker L Silverman B . Increased risk for cancer in young patients with severe obstructive sleep apnea. Respiration. (2019) 97:15–23. doi: 10.1159/000486577 30419556

[12] Tan BKJ Tan NKW Teo YH Yap DW Raghupathy J Gao EY . Association of obstructive sleep apnea with thyroid cancer incidence: a systematic review and meta-analysis. Eur Arch Otorhinolaryngol. (2022) 279:5407–14. doi: 10.1007/s00405-022-07457-w 35708764

[13] Tan BKJ Teo YH Tan NKW Yap DWT Sundar R Lee CH . Association of obstructive sleep apnea and nocturnal hypoxemia with all-cancer incidence and mortality: a systematic review and meta-analysis. J Clin Sleep Med. (2022) 18:1427–40. doi: 10.5664/jcsm.9772 34755597 PMC9059590

[14] Christensen AS Clark A Salo P Nystad W Langhammer A Lowe A . Symptoms of sleep disordered breathing and risk of cancer: a prospective cohort study. Sleep. (2013) 36:1429–35. doi: 10.5665/sleep.3030 24082302 PMC3773192

[15] Hunyor I Cook KM . Models of intermittent hypoxia and obstructive sleep apnea: molecular pathways and their contribution to cancer. Am J Physiol Regul Integr Comp Physiol. (2018) 315:R669–87. doi: 10.1152/ajpregu.00036.2018 29995459

[16] Unnikrishnan D Jun J Polotsky V . Inflammation in sleep apnea: an update. Rev Endocr Metab Disord. (2015) 16:25–34. doi: 10.1007/s11154-014-9304-x 25502450 PMC4346503

[17] Gozal D Almendros I Farré R Wang Y . Obstructive sleep apnea and cancer: epidemiologic links and theoretical biological constructs. Sleep Med Rev. (2016) 27:43–55. doi: 10.1016/j.smrv.2015.05.006 26447849 PMC5374514

[18] Campos-Rodríguez F Martinez-Garcia MA Martinez M Duran-Cantolla J Peña MDL Masdeu MJ . Association between obstructive sleep apnea and cancer incidence in a large multicenter Spanish cohort. Am J Respir Crit Care Med. (2013) 187:99–105. doi: 10.1164/rccm.201209-1671oc 23155146

[19] Nieto FJ Peppard PE Young T Finn L Hla KM Farré R . Sleep-disordered breathing and cancer mortality: results from the Wisconsin Sleep Cohort Study. Am J Respir Crit Care Med. (2012) 186:190–4. doi: 10.1164/rccm.201201-0130oc 22610391 PMC3406081

[20] Eckert DJ Malhotra A . Pathophysiology of adult obstructive sleep apnea. Proc Am Thorac Soc. (2008) 5:144–53. doi: 10.1513/pats.200707-114mg 18250206 PMC2628457

[21] Lavie L . Obstructive sleep apnoea syndrome—an oxidative stress disorder. Sleep Med Rev. (2003) 7:35–51. doi: 10.1053/smrv.2002.0261 12586529

[22] Somers VK Dyken ME Clary MP Abboud FM . Sympathetic neural mechanisms in obstructive sleep apnea. J Clin Invest. (1995) 96:1897–904. doi: 10.1016/0895-7061(96)82092-9 PMC185826 7560081

[23] Lavie L Lavie P . Ischemic preconditioning as a possible explanation for the age decline relative risk of sleep apnea cardiovascular complications. Med Hypotheses. (2006) 66:1069–73. doi: 10.1016/j.mehy.2005.10.033 16513285

[24] Kukwa W Migacz E Druc K Grzesiuk E Czarnecka AM . Obstructive sleep apnea and cancer: effects of intermittent hypoxia? Future Oncol. (2015) 11:3285–98. doi: 10.2217/fon.15.216 26562000

[25] Malhotra A White DP . Obstructive sleep apnoea. Lancet. (2002) 360:237–45. doi: 10.1016/s0140-6736(02)09464-3 12133673

[26] Li L Ren F Qi C Xu L Fang Y Liang M . Intermittent hypoxia promotes melanoma lung metastasis via oxidative stress and inflammation responses in a mouse model of obstructive sleep apnea. Respir Res. (2018) 19:28. doi: 10.1186/s12931-018-0727-x 29433520 PMC5809953

[27] Wang J Chen T Qi X Li Y Yang X Meng X . Retinal vascular fractal dimension measurements in patients with obstructive sleep apnea syndrome: a retrospective case-control study. J Clin Sleep Med. (2023) 19:479–90. doi: 10.5664/jcsm.10370 36458734 PMC9978437

[28] Punjabi NM Caffo BS Goodwin JL Gottlieb DJ Newman AB O'Connor GT . Sleep-disordered breathing and mortality: a prospective cohort study. PLoS Med. (2009) 6:e1000132. doi: 10.1371/journal.pmed.1000132 19688045 PMC2722083

[29] Berry RB Budhiraja R Gottlieb DJ Gozal D Iber C Kapur VK . Rules for scoring respiratory events in sleep: update of the 2007 AASM Manual for the Scoring of Sleep and Associated Events. J Clin Sleep Med. (2012) 8:597–619. doi: 10.5664/jcsm.2172 23066376 PMC3459210

